# Long-Term Changes in Older Adults’ Independence Levels for Performing Activities of Daily Living in Care Settings: A Nine-Year Follow-Up Study

**DOI:** 10.3390/ijerph18189641

**Published:** 2021-09-13

**Authors:** Takuhiro Okabe, Makoto Suzuki, Naoki Iso, Koji Tanaka, Akira Sagari, Hironori Miyata, Gwanghee Han, Michio Maruta, Takayuki Tabira, Masahiro Kawagoe

**Affiliations:** 1 Department of Rehabilitation, Faculty of Health Sciences, Tokyo Kasei University, 2-15-1 Inariyama, Sayama 350-1398, Japan; suzuki-mak@tokyo-kasei.ac.jp (M.S.); iso-n@tokyo-kasei.ac.jp (N.I.); 2 Graduate School of Health Sciences, Gunma University, 3-39-22, Showamachi, Maebashi 371-8514, Japan; kojit929@gunma-u.ac.jp; 3 Department of Occupational Therapy, School of Health Sciences, Faculty of Medicine, Shinshu University, 3-1-1, Asahi, Matsumoto 390-8621, Japan; sagaria@shinshu-u.ac.jp; 4 Department of Rehabilitation, Division of Occupational Therapy, Faculty of Health Science, Kumamoto Health Science University, 325, Izumimachi, Kita-ku, Kumamoto 861-5598, Japan; 814.miya.418@gmail.com; 5 Department of Neuropsychiatry, Kumamoto University Hospital, 1-1-1 Honjo Chuo-ku, Kumamoto 860-8556, Japan; hans11057@gmail.com; 6 Department of Rehabilitation, Medical Corporation, Sanshukai, Okatsu Hospital, 3-95, Masagohonmachi, Kagoshima 890-0067, Japan; m.maru0111@gmail.com; 7 Department of Occupational Therapy, School of Health Sciences, Faculty of Medicine, Kagoshima University, 8-35-1, Sakuragaoka, Kagoshima 890-8544, Japan; tabitaka@health.nop.kagoshima-u.ac.jp; 8 Graduate Course of Health and Social Services, Saitama Prefectural University, 820, Sannomiya, Koshigaya 343-8540, Japan; kawagoe-masahiro@spu.ac.jp

**Keywords:** activities of daily living, aged, longitudinal studies, long-term care, insurance, care prevention

## Abstract

This study aimed to clarify the variability in the independence profiles of specific activities of daily living (ADL) among older men and women. The research subjects were 5872 older adults (1143 men and 4729 women) certified as requiring nursing care or support (based on data obtained from the nursing care insurance certification survey database) who could be surveyed in both 2009 and 2018. Using item response theory, this study compared longitudinal data of difficulties faced by older adults during ADL. The results indicated that among the long-term care insurance-certified persons, in 2009, men had higher ADL difficulty than women in all ADL items, and in 2018, there was no significant difference in items other than dressing and excretion. Furthermore, the difference in the rate of ADL difficulty level over 9 years was significantly higher in women than in men. It was shown that the progression of ADL disability due to aging is faster in men on a yearly basis, but it increases in women with aging. Therefore, it was suggested that the rate of ADL difficulty varies depending on age and sex.

## 1. Introduction

In Japan, over the last few decades, the number of people eligible for long-term care has nearly tripled, from 2.18 million in 2000 to 6.08 million in 2015. [1]. The percentage of older adults who experience difficulties in ADL is as high as 37% in Japan [2]. Owing to the deterioration of mental and physical functions related to aging, older people need support and assistance from others to perform various activities of daily living (ADL) [3]. It is clear that the incidence of disabilities affecting ADL increases with age [3,4]. It has been reported that ADL disorders are caused by age-related deteriorations of physical functioning, pain, and other complications [5]. Research has also found that the degree of independence when performing ADL affects individuals’ quality of life, the prevention of diseases and disorders, and medical costs [4,6]. Accordingly, to ensure the maintenance of individuals’ independence, it is important to understand their degree of independence and provide them with appropriate long-term care prevention strategy. However, the changes that older adults undergo regarding their degree of independence in specific ADL have yet to be analyzed from the perspectives of sex and age.

Japanese people have a relatively long life expectancy, but the difference between average life expectancy and healthy life expectancy (i.e., the number of years that one is unhealthy or has a disability) is 9.13 years for men and 12.68 years for women [7]. Women’s longevity has a significant impact on their health disadvantages [8]. In addition, women’s longer life expectancy also influences men–women differences in health status [9]. It has been shown that the larger women’s longevity excess, the larger their proportion of life in poor health [9]. Such long-lasting disability exerts a heavy burden on health and welfare resources [10]. Nonetheless, interventions to extend healthy life expectancy should not only reduce the financial burden on society but also improve the quality of life for care recipients and their families. Previous studies reported that women account for a higher proportion of people with ADL disabilities than men [11,12,13], but the degree of ADL disability and the rate of disability between men and women who already have ADL disabilities remain unclear. In other words, the rate at which ADL becomes difficult may depend on sex.

In general, physical functionality, especially among dependent older adults, is determined by their ability to undertake ADL, which decreases with age [14]. ADL consist of various undertakings, such as moving, dressing oneself, eating, and bathing. The pattern of changes in older individuals’ ability to undertake these activities is variable [15]. In addition, the contribution of each activity to older adults’ degree of independence differs. Some previous studies have delved into the topic of difficulty performing ADL among older adults. Longitudinal studies have reported the order of occurrence of ADL disorders [16,17,18], while cross-sectional studies have researched their prevalence [19,20,21,22,23]. However, there has been no longitudinal investigation of the long-term changes and sex-based differences in older individuals’ independence levels when undertaking various specific ADL.

This study aimed to (1) assess long-term changes in independence levels, (2) assess the activity-specific independence levels of older adults requiring physical assistance and supervision to perform ADL, and (3) investigate the differences in these matters between men and women. Generating knowledge regarding this progression pattern may aid in the identification of older adult groups at high risk of disability—an important process to ensure the well-informed creation of targeted interventions aimed at maintaining their independence. Clarifying which ADL abilities are maintained and which diminish with age also contributes significantly to long-term care prevention priorities and support priorities. If it is possible to clarify the mode of reduction of ADL disorder among older men and women, it may be possible to contribute to the extension of healthy life expectancy and new strategies for long-term care prevention.

## 2. Materials and Methods

### 2.1. Population, Design

For this retrospective study, we used the 2009–2018 long-term care insurance (LTCI) certification data from City A, Japan. We identified 5872 subjects requiring long-term care who could be tracked for nine years (mean age in 2009: 79.2 ± 6.4 years; 1143 men and 4729 women). Subjects who could not be tracked in 2018 (*n* = 576) were excluded from the analysis. This resulted in a follow-up rate of 91.1% (5872/6448). The LTCI system was implemented by the Japanese government in 2000 to address the accelerating rate of population aging and older adults’ increasing need for care [24]. The LTCI system focuses on long-term care prevention; its main objectives are to enable older adults to live independently in their communities for as long as possible, improve their overall health status, and prevent any physical or mental deteriorations to avoid the need for long-term care [25,26]. The LTCI certification system involves a two-stage process that categorizes individuals into seven levels: support levels 1 and 2 and care needs levels 1 to 5. In the first stage, the required duration of care is estimated by a computer program based on a certification survey. In the second stage, a committee of physicians and other healthcare professionals determines the ranking of individuals based on their care needs. Then, individuals who qualify for long-term care are able to receive the appropriate benefits, while the services provided are divided into two categories: long-term care benefits (care needs levels 1 to 5) and prevention benefits (support levels 1 and 2) [27].

### 2.2. Measurements

Interviews on ADL functions collected data in nine categories: Bathing, Transfer (defined as moving from one flat surface to another; e.g., from a bed to a wheelchair), Mobility, Eating, Urination, Defecation, Grooming (including teeth brushing/mouth rinsing, washing one’s face, and combing/styling one’s hair), Upper-body dressing, and Lower-body dressing. Depending on the degree of assistance required, all items were scored from 1 to 4—except for Grooming, as its items were scored from 1 to 3. Higher scores indicated a lower daily functioning ability in that item and an increased need for assistance [27]. Based on these scores, participants were classified according to whether they were independent (obtaining a score of 1) or not (obtaining a score of 2–4). They were also classified according to their living situation: namely, whether they lived at home (alone or with others) or in an institution. All these assessments were conducted by qualified nursing care investigators (such as doctors, nurses, occupational therapists, social workers, etc.) from the municipalities. Eligibility for LTCI assessment applies to those who are 65 years of age or older and have applied to the municipality. All nine ADL items were evaluated in 2009 and 2018.

### 2.3. Statistical Analysis

A chi-square test was performed with the ADL non-independence rate to determine if there were significant differences for this variable between 2009 and 2018 as well as between men and women. Furthermore, based on item reaction theory (IRT), the data of each item were approximated onto a logistic model, where long-term changes in participants’ independence level for each specific ADL were estimated. IRT can be used to rank items unequivocally along a hierarchy based on their difficulty, as follows:

$$P\left(x\right)=\frac{1}{1+e^{1.7\left(x-\beta \right)}}\;$$

where *x* is a log of the odds of endorsing each activity (i.e., each item), reflecting participants’ disability level. *β*, which is the coefficient of IRT, is a parameter of the difficulty level and is based on 0. The difficulty level becomes lower in the negative direction and higher in the positive direction. Additionally, *β* is a curve that shifts from the left to the right as the value increases, reflecting the severity of the disability for each activity. The difference between *β* in 2009 and 2018 was calculated, and the difference between men and women was examined by a nonparametric test. All statistical analyses were performed using R 3.5.2 software (R Foundation for Statistical Computing, Vienna, Austria).

### 2.4. Ethical Considerations

Saitama Prefectural University and municipal government signed the memorandum of data handling according to the local government regulation for privacy policy. Additionally, the Ethics Committee of Saitama Prefectural University approved the procedure of this study with anonymized database (No. 20019) in 17 June 2020.

## 3. Results

Table 1 shows participants’ characteristics. In the 2009 sample, the group of participants requiring support level 2 was the largest, followed by those requiring long-term care level 1, and the smallest being those requiring long-term care level 5. Conversely, in the 2018 sample, the group of participants requiring long-term care level 4 was the largest, which was followed by participants requiring long-term care level 3, and the smallest group comprised individuals requiring support level 2. In 2009, the group of individuals requiring support level 1 regarding nursing care tended to be large; however, in 2018, the proportion of individuals requiring nursing care in levels 2 to 5 increased.

Table 2 shows each sample’s (2009 or 2018) non-independence rate for specific ADL. In the 2009 sample, Grooming (58.6%) had the highest non-independence rate, which was followed by Bathing (49.7%), Upper-body dressing (29.4%), Lower-body dressing (28.6%), Mobility (25.4%), Urination (25.1%), Defecation (23.3%), Transfer (18.6%), and Eating (12.0%). The aforementioned top and bottom three items were the same for all subjects, regardless of sex. In addition, the ranking of specific ADL by non-independence rates was similar between the overall sample and women; however, the ranking of the three middle items was different for men.

In the 2018 sample, Grooming (89.8%) still had the highest non-independence rate, which was followed by Bathing (85.1%), Upper-body dressing (68.4%), Urination (66.2%), Defecation (64.0%), Lower-body dressing (62.9%), Mobility (62.2%), Transfer (49.9%), and Eating (9.0%). Specifically, owing to aging, specific ADL non-independence rates were significantly higher in 2018 than in 2009 for all subjects, regardless of sex. The non-independence rate of all specific ADL among men was higher than that among women in 2009 (*p* < 0.0001 for all items). Contrarily, in 2018, the non-independent rate of Upper-body dressing, Lower-body dressing, Urination, and Defecation in men was significantly higher, and there was no difference between men and women in other specific ADL items.

In 2009, men had a significantly higher rate of non-independence in ADL than women in all items. Contrarily, in 2018, men had a higher rate of non-independence than women only in Upper-body dressing, Lower-body dressing, Urination, and Defecation.

**Figure 1:**
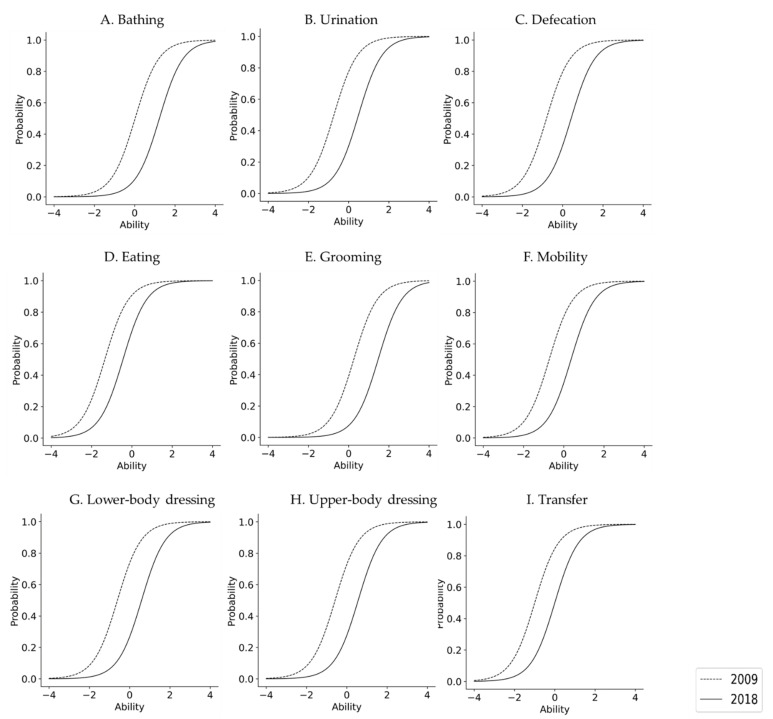
Difficulties in their ADL independence for all participants from 2009 to 2018.

**Figure 2:**
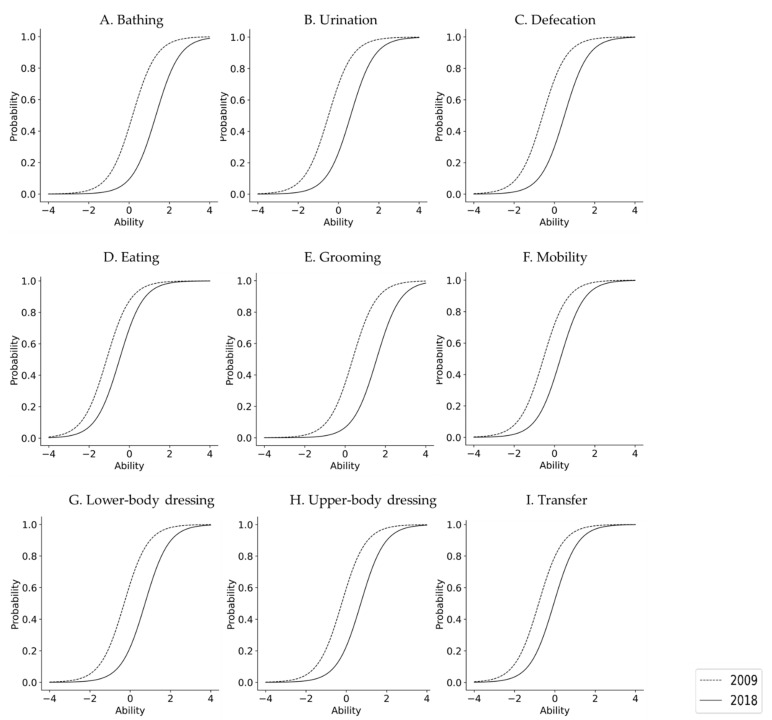
Difficulties in their ADL independence for men from 2009 to 2018.

**Figure 3:**
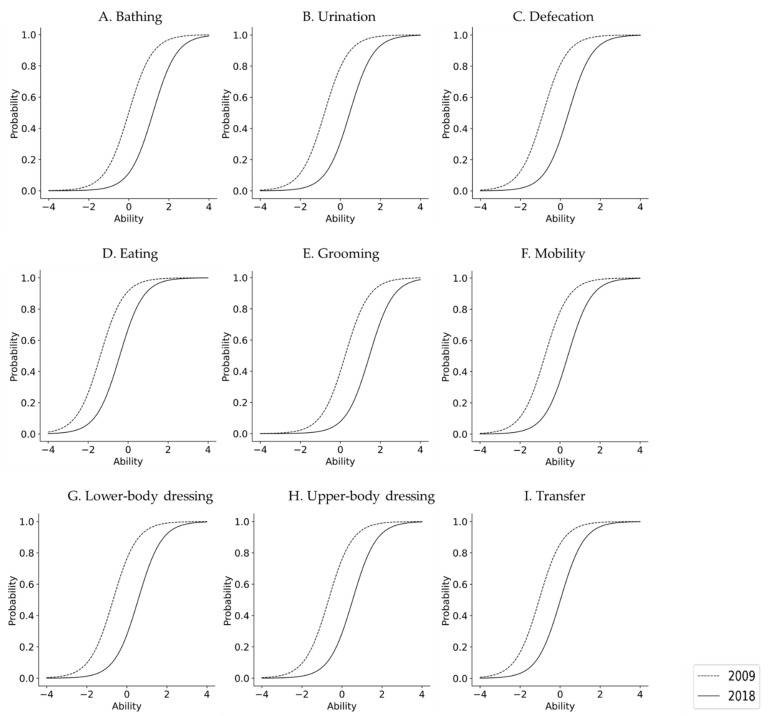
Difficulties in their ADL independence for women from 2009 to 2018.

The IRT results are shown in Table 3. In both 2009 and 2018, all items were compatible with the logistic model (*p* < 0.0001 for all items). In terms of IRT, ADL difficulty was significantly higher in 2018 than in 2009 for all subjects, regardless of sex. In 2009, for the overall sample and for women, the difficulty level in descending order (highest to lowest) was as follows: Grooming, Bathing, Upper-body dressing, Lower-body dressing, Mobility, Urination, Defecation, Transfer, and Eating. For men, the same ranking was as follows: Grooming, Bathing, Upper-body dressing, Lower-body dressing, Urination, Mobility, Defecation, Transfer, and Eating. In 2018, nonetheless, this ranking for the overall sample was as follows: Grooming, Bathing, Lower-body dressing, Upper-body dressing, Urination, Defecation, Mobility, Transfer, and Eating. In other words, the rankings across the three groups were the same in the second assessment period. Furthermore, the difference in β between 2009 and 2018, that is, the rate of increase in difficulty level, was significantly higher in women than in men. In 2018, the curve of the model was displaced to the right in all items when compared to 2009, with the probability of independence having decreased (Figure 1, Figure 2 and Figure 3).

The X-axis denotes each person’s disability level. The Y-axis denotes the probability of independence for each activity. The dotted line represents the 2008 sample, and the solid line represents the 2018 sample. In 2018, the curve of the model shifted to the right, meaning that the difficulty increased for all items when compared to 2009.

Similarly for men, in 2018, the curve of the model shifted to the right, meaning that the difficulty increased for all items when compared to 2009. Both years were generally shifted to the right, suggesting that ADL difficulty is high in men. The changes in 2009 and 2018 were particularly remarkable in grooming, bathing, urination, and defecation, but the changes in other items were relatively small.

Similarly for women, in 2018, the curve of the model shifted to the right, meaning that the difficulty increased for all items when compared to 2009. Similar to men, the changes in 2009 and 2018 were particularly remarkable in grooming, bathing, urination, and defecation, but it was suggested that the changes in each item were larger than in men.

## 4. Discussion

This study corroborated that the proportion of people who had difficulty remaining independent in their ADL increased with age in both men and women. Past studies have shown that the pattern of ADL disabilities in geriatric populations follows a distinct progression [16,17,18,21,22,28]. Among those certified for long-term care insurance, although the average age of men was lower than that of women, the proportion of men with difficulties in their ADL independence was higher than that of women. In short, among people with ADL disorders, ADL were more difficult for men than for women. This result contradicts previous studies that women have a higher prevalence in this regard [11,12,13]. The reason for the lower independence level in men is that the previous study recruited from the general population of older people, whereas our study focused on older people who have difficulty in ADL due to mental or physical disorder (i.e., recruiting from the persons eligible for the LTCI system). In this study, in 2009, men had a higher rate of non-independence than women in all ADL items among those who were certified as requiring nursing care. In 2018, only dressing and excretion difficulties were higher in men than in women. Furthermore, the difference in β over 9 years was significantly larger in women than in men. In other words, it was shown that the progression of ADL disorder due to aging is faster in men on a yearly basis but increases in women with aging. It is suggested that men with ADL disorder have many other ADL disabilities from the beginning, and that once women have ADL disability, their ADL ability declines sharply with aging. This suggests that comprehensive ADL support may be needed for men, and local and staged interventions may be needed for women when intervening for older adults who need care. Sauvaget et al. reported that the development and progression of disability were different between sexes: men experienced disability at a younger age and faster rate than women [10]. The slow progress of disability, with a longer duration in a disabled state among women, induces a heavy burden on health and welfare resources [10]. In this study, the younger average age of men showed the difference in lifespan between men and women, and it confirmed the paradox, “men die early and women suffer for a long time”, as stated in a previous study [29]. This is likely because, in general, life expectancy is longer in women than in men, with the likelihood of women being separated from their spouses being higher than that of men. Kishimoto et al. reported that support for family members becomes necessary in Japan when a man experiences difficulties in ADL because Japanese husbands are highly dependent on their wives in performing these activities [20]. Conversely, women are expected to be more independent than men in their ADL because Japanese wives cannot depend on family members, especially their husbands. This is because husbands tend to typically not help their wives in their ADL [20,30]. Kuzuya et al. reported that women had a higher rate of living alone and a lower rate of receiving care by a spouse [31]. It must be recognized that after age 75, women are more likely to be widowed, while older men are more likely to have a wife who can assist them in the event of disability [32]. In this study, nine years later, women retained their ability to dress themselves and excrete. A previous study reported that women in nursing homes remained independent longer for toilet use [33]. For this reason, women may be particularly reluctant to being assisted with changing clothes and excreting. Therefore, an individual’s transition of ADL independency differs depending on their daily lifestyle, environment, and individual role in the home. There are a few explanations why women live longer than men but have poorer health. Several studies exhibit that this is due to biological, social, and behavioral factors [34,35,36]. Researchers have suggested that two factors in particular contribute to this phenomenon. First, women are more likely than men to adopt preventative health behaviors, such as routine annual visits to a physician for a check-up. Studies argue that poorer health among women is due to biological as well as behavioral factors. Some studies discuss that while women suffer more than men, women’s ailments tend to be less lethal biologically [37,38]. Some other studies refer to the over-reporting of worse health among women [39,40].

This study’s results suggest that the order of independence probabilities according to each activity item is generally the same, although the independence levels of various activities in ADL do decrease over the long term. Shinkai et al. reported that the number of people with disabilities increases with age, which corroborates the current study’s findings [4]. This study demonstrated that difficulties in ADL among older adults significantly increase after nine years. We also discovered that the order of difficulty in ADL was highest for Grooming and Bathing and was lowest for Eating and Transfer, which remained unchanged after nine years. Previous cross-sectional [19,20,21,23] and longitudinal studies [17,18] have reported that bathing and eating tend to be challenging for older adults with ADL difficulties. This was consistent with the results of the present study. Bathing is a complex task involving multiple subtasks, such as undressing and drying one’s body [41]. The incidences of bathing-related disabilities (defined as needing personal assistance with this activity) per 1000 person-months is as high as 23.0 for those aged 70 to 79 years and 43.6 for those aged 80 years or older [42]. Bathing disability has been independently associated with an increased likelihood of long-term nursing home admission [43] and is a primary indicator of the need for home care services [44]. Japanese people generally prefer to take baths rather than taking showers or sauna baths not only for cleanliness but also to feel warm and refreshed as well as to aid in sleep [45]. Morris et al. reported that individuals receiving home care in Hong Kong had the highest rate of grooming disabilities [22]. This is consistent with this study’s findings. However, there are few reports on grooming, with the order of difficulties often being different [19]. Typically, grooming, bathing, and dressing are considered low priority as activities needed to maintain life, unlike eating, moving, and using the toilet. Therefore, older adults with some disabilities may be less interested in grooming and bathing and therefore less likely to undertake these activities. In addition, when providing support to older adults with disabilities within a limited timeframe, the main interventions provided are for Eating, Transfer, and Mobility, but Grooming, Bathing, and Dressing tend to be overlooked by carers and healthcare practitioners. Even if an intervention is provided, it often does not make the most of the care recipient’s remaining ability, with the amount of assistance given being high. However, tasks involving dressing and keeping clean support one’s sense of dignity and are important regardless of a person’s age or disability [46]. Dubuc et al. reported that grooming and bathing do not specifically meet the needs of community-dwelling older adults with disabilities [32]. Thus, for these two activities, which do tend to be more difficult, we need to consider ways in which to provide further support in terms of care prevention. Our findings provide useful information for developing a care prevention plan to maintain the independence of older adults. Both longitudinal and cross-sectional studies agree that bathing deficits precede feeding ones; however, there is less agreement on the sequence of the development of other disabilities, such as those surrounding grooming, dressing, urination, defecation, mobility, and transfer, either due to differences in the analytic techniques used or due to a wide heterogeneity in the sequence in which functions are lost over time [16,17,18,19,23]. Several studies have also highlighted the fact that disability progression differs by sex [16], time period [28], institutional settings (e.g., residential setting or nursing homes) [28], types of ADL items [21], and countries [47]. Thus, when deciding on whether to assist someone with ADL difficulties, we must consider the subject’s life history and culture, their sex, and living environment. In addition to the important items needed for maintaining life, such as eating, moving, and walking, care should be taken to maintain each individual’s sense of dignity through cleanliness and grooming.

This study has some limitations. First, the findings were limited to residents within the LTCI system and therefore, they may reflect the idiosyncratic nature of those receiving treatment, nursing care, and functional assessments within one large healthcare system. In order to generalize this study’s findings, the survey should be expanded to general older adults and middle-aged people. Second, in this study, the factors of difficulty for each ADL item were not clarified. Further research is needed to clarify these factors in terms of the order of difficulty of each item.

## 5. Conclusions

This study reveals that among the long-term care insurance certified persons, in 2009, men had higher ADL difficulty than women in all ADL items, and in 2018, there was no significant difference in items other than changing clothes and excretion. Furthermore, the difference in β over 9 years was significantly larger in women than in men. In other words, it was shown that the progression of ADL disability due to aging is faster in men on a yearly basis, but it increases in women with aging. It was suggested that men with ADL disorder had many other ADL disabilities from the beginning, and that once women had an ADL disorder, their ADL ability declined sharply with aging. This suggestion may indicate that we need comprehensive ADL support for men and local and staged interventions for women when providing interventions for older adults in need of care. Furthermore, the difficulty levels were relatively low for Eating and Transfer and were high for Grooming and Bathing. Therefore, our study results will assist healthcare practitioners in understanding and anticipating specific target areas for interventions aimed at older adults. In other words, the findings from this study provide useful information for the development of effective care prevention plans that aim to maintain the independence of older adults.

## Figures and Tables

**Table 1:** Participants’ characteristics (*N* = 5872).

	2009	2018
	Mean ± SD, *n* (%)	
Sex	Men 1143 (19.5)	
	Women 4729 (80.5)	
Age (years)	79.2 ± 6.4	88.2 ± 6.4
	Men 76.2 ± 6.3	Men 85.2 ± 6.3
	Women 80.0 ± 6.2	Women 89.0± 6.2
Lived at home	5451 (92.8)	4542 (77.4)
Living alone	2497 (42.5)	2829 (48.2)
Living together	2954 (50.3)	1713 (29.2)
In an institution	421 (7.2)	1330 (22.6)
Care-need level		
Requiring support 1	1050 (17.9)	247 (4.2)
Requiring support 2	1816 (30.9)	624 (10.6)
Requiring long-term care 1	1285 (21.9)	718 (12.2)
Requiring long-term care 2	807 (13.7)	1024 (17.4)
Requiring long-term care 3	452 (7.7)	1039 (17.7)
Requiring long-term care 4	304 (5.2)	1211 (20.6)
Requiring long-term care 5	158 (2.7)	1009 (17.2)

SD: Standard deviation.

**Table 2:** Non-independency prevalence rates for ADL items in 2009 and 2018.

					*n* = 5872
	ADL Items	All	Men	Women	
		% (*n*)	% (*n*)	% (*n*)	*p* ^†^
2009 y	Grooming	58.6 (3443)	63.8 (729)	57.4 (2714)	<0.001
	Bathing	49.7 (2921)	57.1 (653)	48.0 (2268)	<0.001
	Upper-body dressing	29.4 (1724)	43.2 (494)	26.0 (1230)	<0.001
	Lower-body dressing	28.6 (1680)	40.8 (466)	25.7 (1214)	<0.001
	Mobility	25.4 (1490)	32.7 (374)	23.6 (1116)	<0.001
	Urination	25.1 (1476)	34.6 (395)	22.9 (1081)	<0.001
	Defecation	23.3 (1366)	31.9 (365)	21.2 (1001)	<0.001
	Transfer	18.6 (1094)	25.5 (291)	17.0 (803)	<0.001
	Eating	12.0 (703)	17.4 (199)	10.7 (504)	<0.001
2018 y	Grooming	89.8 (5275)	90.6 (1035)	89.7 (4240)	0.383
	Bathing	85.1 (5000)	86.8 (992)	84.8 (4008)	<0.086
	Upper-body dressing	68.4 (4017)	72.9 (833)	67.3 (3184)	<0.001
	Lower-body dressing	62.9 (4064)	73.4 (839)	68.2 (3225)	<0.001
	Mobility	62.2 (3654)	60.0 (686)	62.8 (2968)	0.089
	Urination	66.2 (3885)	69.6 (796)	65.3 (3089)	0.006
	Defecation	64.0 (3757)	66.6 (761)	63.4 (2996)	0.043
	Transfer	49.9 (2933)	48.1 (550)	50.4 (2383)	0.177
	Eating	34.9 (2051)	32.9 (376)	35.4 (1675)	0.112
					χ^2^ test

^†^ Significance of the difference in the ADL items’ non-independency between men and women. ADL: activities of daily living.

**Table 3:** Proportion of positive responses for each ADL item in both 2009 and 2018.

		2009 y					2018 y					Δβ
	ADL Items	β_2009_	SE	χ^2^	*p*	R	β_2018_	SE	χ^2^	*p*	R	
All	Grooming	0.263	0.018	398.5	<0.001	1	1.476	0.025	376.3	<0.001	1	1.213
	Bathing	0.025	0.016	207.2	<0.001	2	1.231	0.025	393.2	<0.001	2	1.206
	Upper-body dressing	−0.578	0.018	86.8	<0.001	3	0.568	0.020	205.8	<0.001	4	1.146
	Lower-body dressing	−0.604	0.018	153.9	<0.001	4	0.595	0.021	220.2	<0.001	3	1.199
	Mobility	−0.721	0.018	77.9	<0.001	5	0.365	0.020	1112.8	<0.001	7	1.086
	Urination	−0.73	0.018	188.1	<0.001	6	0.493	0.020	116.1	<0.001	5	1.223
	Defecation	−0.802	0.019	216.8	<0.001	7	0.421	0.020	141.8	<0.001	6	1.223
	Transfer	−0.998	0.020	49.3	<0.001	8	0.0	0.019	1746.2	<0.001	8	0.998
	Eating	−1.338	0.022	189.8	<0.001	9	−0.436	0.022	1493.2	<0.001	9	0.902
Men	Grooming	0.376	0.046	16.6	<0.001	1	1.564	0.059	61.2	<0.001	1	1.188
	Bathing	0.177	0.044	25.7	<0.001	2	1.335	0.054	68.6	<0.001	2	1.158
	Upper-body dressing	−0.226	0.042	22.3	<0.001	3	0.72	0.046	27.9	<0.001	4	0.946
	Lower-body dressing	−0.299	0.043	44.4	<0.001	4	0.739	0.046	56.2	<0.001	3	1.038
	Mobility	−0.552	0.044	19.0	<0.001	6	0.293	0.043	50.2	<0.001	7	0.845
	Urination	−0.493	0.044	62.6	<0.001	5	0.605	0.045	35.2	<0.001	5	1.098
	Defecation	−0.578	0.045	69.3	<0.001	7	0.501	0.044	34.8	<0.001	6	1.079
	Transfer	−0.803	0.047	20.3	<0.001	8	−0.049	0.043	21.7	<0.001	8	0.754
	Eating	−1.131	0.052	37.7	<0.001	9	−0.49	0.046	89.7	<0.001	9	0.641
Women	Grooming	0.241	0.019	447.4	<0.001	1	1.458	0.027	433.1	<0.001	1	1.217
	Bathing	−0.005	0.017	214.0	<0.001	2	1.21	0.027	433.9	<0.001	2	1.215
	Upper-body dressing	−0.669	0.021	68.6	<0.001	3	0.538	0.022	248.5	<0.001	4	1.207
	Lower-body dressing	−0.681	0.021	108.9	<0.001	4	0.567	0.022	221.3	<0.001	3	1.248
	Mobility	−0.76	0.021	133.4	<0.001	5	0.388	0.021	780.1	<0.001	7	1.148
	Urination	−0.789	0.021	138.4	<0.001	6	0.472	0.022	131.0	<0.001	5	1.261
	Defecation	−0.859	0.022	160.1	<0.001	7	0.407	0.021	166.6	<0.001	6	1.266
	Transfer	−1.049	0.024	112.7	<0.001	8	0.017	0.021	1564.1	<0.001	8	1.066
	Eating	−1.394	0.026	188.6	<0.001	9	−0.421	0.024	1270.7	<0.001	9	0.973
		Men median (quartile)					Women median (quartile)					*p* ^†^
Total of Δβ		1.038 (0.845, 1.098)					1.215 (1.148, 1.248)					0.008

ADL: activities of daily living, β: item severity, SE: standard error, R: rank based on β, Δβ: difference between β2009 and β2018. ^†^ Significance of the difference in total of Δβ between men and women.
